# Author Correction: Independent fitness consequences of group size variation in Verreaux’s sifakas

**DOI:** 10.1038/s42003-024-06611-w

**Published:** 2024-08-02

**Authors:** Peter M. Kappeler, Claudia Fichtel

**Affiliations:** 1 Behavioral Ecology & Sociobiology Unit, German Primate Center, Leibniz Institute for Primate Research, Kellnerweg 4, 37077 Göttingen, Germany; 2 Department of Sociobiology/Anthropology, Johann-Friedrich-Blumenbach Institute of Zoology and Anthropology, University Göttingen, Kellnerweg 6, 37077 Göttingen, Germany

Correction to: *Communications Biology* 10.1038/s42003-024-06484-z, published online 05 July 2024

In the original version of the Article, the y-axis of Figure 4 was erroneously labelled “Annual survival”. This has been corrected to “Probability of dying” in the updated Article.

