# Supplementary material for: Independent fitness consequences of group size variation in Verreaux’s sifakas
Source: Commun Biol. 2024 Jul 5;7:816. doi: 10.1038/s42003-024-06484-z (PMC11224245; doi:10.1038/s42003-024-06484-z)
Supplement: Supplementary file 2 — Supplementary Information [file 42003_2024_6484_MOESM2_ESM.docx]

**Additional information**

**Supplementary information**

**Independent fitness consequences of group size variation in Verreaux’s sifakas**

Peter M. Kappeler and Claudia Fichtel

****************************************************************

**Table S1**. Summary of the results of the models estimating the probability of giving birth including a) the number of adult females, b) ASR, and c) all disappearances.

| **Models** | **Terms** | **Estimate** | **Std. Error** | **P** |
| --- | --- | --- | --- | --- |
| **a) birth rates** | Intercept | 0.90 | 0.16 | ^a^ |
| **including number** | Group size | 0.33 | 0.20 | 0.099 |
| **of adult females** | Female age | 0.48 | 0.15 | 0.002 |
|  | Rainfall | -0.03 | 0.19 | 0.87 |
|  | N females | -0.94 | 0.27 | <0.001 |
| **b) birth rates** | Intercept | 1.40 | 0.25 | ^a^ |
| **including ASR** | Group size^b^ | -0.20 | 0.16 | 0.219 |
|  | Group size^2b^ | -0.10 | 0.11 | 0.415 |
|  | Female age^b^ | 0.64 | 0.16 | <0.001 |
|  | Female age^2b^ | -0.43 | 0.17 | 0.014 |
|  | Rainfall^b^ | -0.23 | 0.21 | 0.275 |
|  | ASR^b^ | -0.10 | 0.15 | 0.500 |
| **c) annual survival** | Intercept | -2.35 | 0.33 | ^a^ |
| **including all** | Group size^b^ | 0.83 | 0.21 | <0.001 |
| **disappearances** | Group size^2b^ | 0.12 | 0.18 | 0.474 |
|  | Individual age^b^ | -0.77 | 0.14 | <0.001 |
|  | Individual age^2b^ | 0.94 | 0.16 | <0.001 |
|  | Rainfall^b^ | 0.00 | 0.19 | 0.987 |
|  | ASR^b^ | -0.16 | 0.16 | 0.288 |
| ^a^ Not shown as has no meaningful interpretation | |  |  |  |
| ^b^ z-transformed to mean of zero and a standard deviation of one; mean and standard deviation of the orginal predictors: | | | | |
| model a, b): group size: 6.26±1.96; female's age: 9.44±4.37; N females: 1.93±0.84; rainfall: 894.72±175.24; ASR:0.52±0.13 | | | | |
| model c): group size: 5.98±1.91; individual's age: 6.38±5.15; N females: 1.93±0.84; rainfall: 866.02±176.77; ASR:0.56±0.12 | | | | |

**Table S2**: Summary of the results of the models estimating the probability of giving birth including a) quadratic terms, b) linear terms and ASR, c) linear terms, dominance status (yes, no) and the number of adult females, d) quadratic terms, dominance status (yes, no) and the number of adult females, e) linear terms, dominance status (yes, no) and ASR, and f) quadratic terms, dominance status (yes, no) and ASR.

| **Models** | **Terms** | **Estimate** | **Std. Error** | **P** |
| --- | --- | --- | --- | --- |
| **a) quadratic terms:** | Intercept | 1.29 | 0.25 | ^a^ |
| **birth rates** | Group size | 0.24 | 0.22 | 0.264 |
| **including number** | Group size^2b^ | -0.11 | 0.13 | 0.405 |
| **of adult females** | Female age | 0.46 | 0.17 | 0.006 |
|  | Femaleage^2^ | -0.39 | 0.18 | 0.030 |
|  | Rainfall | -0.06 | 0.20 | 0.767 |
|  | N females | -0.88 | 0.29 | 0.003 |
|  | N females^2^ | 0.08 | 0.14 | 0.557 |
|  | *likelihood ratio full-null model comparison: x^2^=33.38, df=6, p<0.001* | | | |
|  | *AIC: model incl. linear terms=425.99, model incl. squared terms=434.96* | | | |
| **b) linear terms:** | Intercept | 0.95 | 0.20 | ^a^ |
| **birth rates** | Group size | -0.19 | 0.15 | 0.205 |
| **including ASR** | Female age | 0.76 | 0.22 | <0.001 |
|  | Rainfall | -0.20 | 0.20 | 0.334 |
|  | ARS | -0.02 | 0.14 | 0.868 |
|  | *likelihood ratio full-null model comparison: x^2^=14.32, df=3, p=0.002* | | | |
|  | *AIC: model incl. linear terms=441.07, model incl. squared terms=440.17* | | | |
| **c) linear terms:** | Intercept | 0.52 | 0.33 | ^a^ |
| **birth rates** | Group size | 0.28 | 0.24 | 0.248 |
| **including number** | Female age | 0.41 | 0.28 | 0.134 |
| **of adult females** | Rainfall | -0.02 | 0.23 | 0.914 |
| **and dominance** | N females | -0.98 | 0.53 | 0.066 |
| **status** | Dominance^b^ | 0.62 | 0.44 | 0.160 |
|  | *likelihood ratio full-null model comparison: x^2^=18.45, df=4, p=0.001* | | | |
| **d) quadratic terms:** | Intercept | 0.88 | 0.41 | ^a^ |
| **birth rates** | Group size | 0.27 | 0.28 | 0.326 |
| **including number** | Group size^2^ | -0.18 | 0.22 | 0.412 |
| **of adult females** | Female age | 0.21 | 0.27 | 0.445 |
| **and dominance** | Female age^2^ | -0.60 | 0.21 | 0.004 |
| **status** | Rainfall | -0.05 | 0.22 | 0.821 |
|  | N females | 0.75 | 1.36 | 0.579 |
|  | N females^2^ | -0.87 | 0.65 | 0.182 |
|  | Dominance^b^ | 0.68 | 0.49 | 0.166 |
|  | *likelihood ratio full-null model comparison: x^2^=26.01, df=7, p<0.001* | | | |
|  | *AIC: model incl. linear terms=441.07, model incl. squared terms=440.17* | | | |
| **e) linear terms:** | Intercept | 0.31 | 0.33 | ^a^ |
| **birth rates** | Group size | 0.08 | 0.33 | 0.713 |
| **including ASR** | Female age | 0.82 | 0.33 | 0.012 |
| **and dominance** | Rainfall | -0.14 | 0.24 | 0.574 |
| **status** | ARS | -0.40 | 0.23 | 0.087 |
|  | Dominance^b^ | 0.67 | 0.43 | 0.119 |
|  | *likelihood ratio full-null model comparison: x^2^=32.40, df=6, p<0.001* | | | |
| **f) quadratic terms:** | Intercept | 0.82 | 0.36 | ^a^ |
| **birth rates** | Group size | 0.46 | 0.33 | 0.162 |
| **including ASR** | Group size^2^ | -0.41 | 0.18 | 0.041 |
| **and dominance** | Female age | 0.35 | 0.24 | 0.142 |
| **status** | Female age^2^ | -0.64 | 0.18 | <0.001 |
|  | Rainfall | -0.13 | 0.24 | 0.576 |
|  | ASR | -0.48 | 0.28 | 0.088 |
|  | Dominance^b^ | 0.86 | 0.46 | 0.058 |
|  | *likelihood ratio full-null model comparison: x^2^=32.40, df=6, p<0.001* | | | |
|  | *AIC: model incl. linear terms=324.28, model incl. squared terms=320.75* | | | |
| ^a^ Not shown as has no meaningful interpretation | | |  |  |
| ^b^ dominance status (yes) | |  |  |  |

**Table S3:** Summary of the results of the models estimating annual survival including a) quadratic terms and all disappearances, b) linear terms and only confirmed deaths, c) quadratic terms and only confirmed deaths, d) linear terms and confirmed deaths as well as all females with unknown disappearance, e) quadratic terms and confirmed deaths as well as all females with unknown disappearance.

| **Models** | **Terms** | **Estimate** | **Std. Error** | **P** |
| --- | --- | --- | --- | --- |
| **a) linear terms:** | Intercept | -1.34 | 0.30 | ^a^ |
| **annual survival** | Group size | 0.69 | 0.19 | <0.001 |
| **including all** | Individual age | -1.23 | 0.11 | <0.001 |
| **disappearances** | Rainfall | -0.12 | 0.17 | 0.486 |
|  | ASR | -0.14 | 0.14 | 0.336 |
|  | *likelihood ratio full-null model comparison: x^2^=46.18, df=3, p<0.001* | | | |
|  | *AIC: model incl. linear terms=818.68, model incl. squared terms=764.28* | | | |
| **b) linear terms:** | Intercept | -2.99 | 0.27 | ^a^ |
| **annual survival** | Group size | 0.39 | 0.20 | 0.054 |
| **including confirmed** | Individual age | -1.53 | 0.15 | <0.001 |
| **deaths** | Rainfall | 0.01 | 0.13 | 0.912 |
|  | ASR | -0.14 | 0.15 | 0.349 |
|  | *likelihood ratio full-null model comparison: x^2^=10.02, df=3, p=0.018* | | | |
| **c) quadratic terms:** | Intercept | -3.00 | 0.33 | ^a^ |
| **annual survival** | Group size | 0.83 | 0.29 | 0.028 |
| **including confirmed** | Group size^2^ | 0.04 | 0.19 | 0.578 |
| **deaths** | Individual age | -0.40 | 0.16 | 0.030 |
|  | Individual age^2^ | 1.16 | 0.17 | <0.001 |
|  | Rainfall | -0.08 | 0.20 | 0.396 |
|  | ASR | -0.27 | 0.15 | 0.255 |
|  | *likelihood ratio full-null model comparison: x^2^=118.34, df=5, p<0.001* | | | |
|  | *AIC: model incl. linear terms=513.42, model incl. squared terms=407.14* | | | |
| **d) linear terms:** | Intercept | -1.77 | 0.24 | ^a^ |
| **annual survival** | Group size | 0.61 | 0.19 | 0.002 |
| **including confirmed** | Individual age | -1.04 | 0.11 | <0.001 |
| **deaths and females** | Rainfall | -0.19 | 0.18 | 0.279 |
| **with unknown** | ASR | -0.19 | 0.11 | 0.090 |
| **disappearance** | *likelihood ratio full-null model comparison: x^2^=44.28, df=3, p<0.001* | | | |
| **e) quadratic terms:** | Intercept | -3.00 | 0.33 | ^a^ |
| **annual survival** | Group size | 0.83 | 0.29 | 0.005 |
| **including confirmed** | Group size^2^ | 0.04 | 0.19 | 0.818 |
| **deaths and females** | Individual age | -0.40 | 0.16 | 0.010 |
| **with unknown** | Individual age^2^ | 1.16 | 0.17 | <0.001 |
| **disappearance** | Rainfall | -0.08 | 0.20 | 0.697 |
|  | ASR | -0.27 | 0.15 | 0.081 |
|  | *likelihood ratio full-null model comparison: x^2^=49.19, df=5, p<0.001* | | | |
|  | *AIC: model incl. linear terms=771.37, model incl. squared terms=685.47* | | | |
| ^a^ Not shown as has no meaningful interpretation | |  |  |  |
